# A rare complication following internal jugular vein catheterization to malposition: acute Budd Chiari syndrome

**DOI:** 10.1186/s12882-020-02182-0

**Published:** 2020-12-03

**Authors:** Sumeyra Koyuncu, Nevzat Herdem, Cihan Uysal, Guven Kahriman, Ismail Kocyigit, Murat Sipahioğlu, Bulent Tokgoz, Oktay Oymak

**Affiliations:** 1 Department of Internal Medicine, Division of Nephrology, Erciyes University School of Medicine, Kayseri, Turkey; 2 Department of Radiology, Erciyes University School of Medicine, Kayseri, Turkey

**Keywords:** Budd Chiari, Catheter, Hemodialysis, Malposition

## Abstract

**Background:**

Tunneled catheters can be used as an alternative vascular access in patients with limited health expectancy,vascular access problems and several comorbidities. We aimed to present a patient with venous stenosis related- reversible acute Budd-Chiari syndrome after catheter malposition.

**Case presentation:**

After changing of tunneled catheter insertion, 36-year old man was admitted to our hospital with sudden onset of nausea, fever, chills and worsening general condition In computed tomography (CT) imaging, a hypodense thrombus was observed in which the distal end of the catheter is at the level of drainage of the hepatic veins in the inferior vena cava and that blocked hepatic vein drainage around the catheter. The catheter was removed and a new catheter was inserted in the same session. Because patient’s general condition was good and without fever, he was discharged with advices on the 9th day of hospitalization.

**Conclusion:**

Although catheter malposition and thrombosis are not a common complication, clinicians should be alert of these complications.

## Background

The incidence of end stage renal disease (ESRD), which is one of the important health problems of worldwide, is gradually increasing and one of the treatment options is hemodialysis. Hemodialysis requires effective vascular access routes with a blood flow of at least 350 mL/min [1–3]. It has been suggested that the first choice for vascular access is arteriovenous fistula (AVF), but tunneled catheters can be used as an alternative to AVF in patients with limited health expectancy, comorbidities and vascular access problems according to Kidney Disease Outcomes Quality Initiative (KDOQI) guidelines [4].

Infection, internal jugular vein (IJV) stenosis and thrombosis are also common complications of permanent intrajugular hemodialysis catheterization [5]. Despite all complications, usage of dialysis catheter is still very common in this cohort according to the annual report of the United States Renal Data System (USRDS), approximately 63% of patients used a catheter for vascular access for the first dialysis treatment in the USA [6]. Another important complication such as catheter malposition ratio was between 3.6–14% in small and medium-sized studies in the literature. However, catheter malposition is rare in right IJV catheterization [7, 8].

Additionally, all complications related to IJV (infection, hematoma, malposition) were found to have a lower rate in the literature [9–11].

Budd-Chiari syndrome is caused by partial/complete hepatic venous perfusion obstruction at any localization of hepatic vein, inferior vena cava or right atrium level. As a result, liver venous obstruction occurs due to increased hepatic sinusoidal pressure, which leads to portal hypertension and decreased liver perfusion. Patients can usually apply with ascites, abdominal swelling and pain. This process may eventually progress to liver fibrosis and cirrhosis [12, 13].

In this case report, we aimed to present a patient with venous stenosis related- reversible acute Budd-Chiari syndrome after catheter malposition.

## Case presentation

A 36-year-old man was admitted to the emergency department of our university hospital with sudden onset of nausea, fever, chills and worsening general condition.

**Fig. 1:**
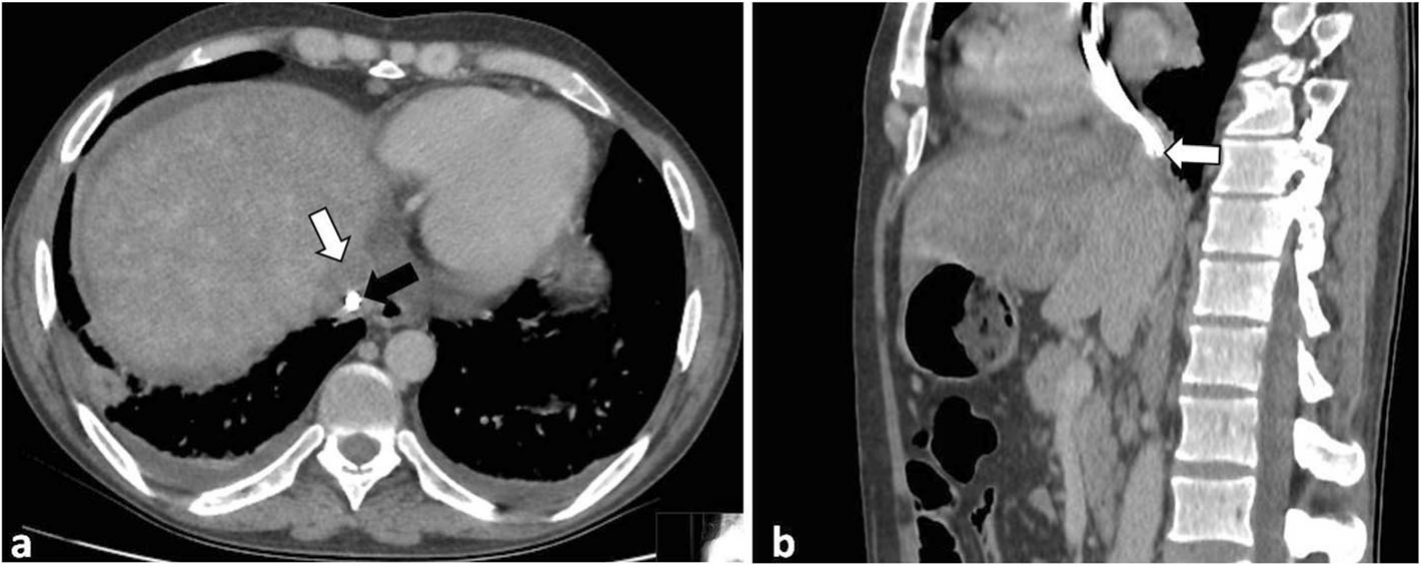
**a** and **b** In axial contrast venous phase CT section (**a**), the distal end of the dialysis catheter is located at the level of drainage of the hepatic veins in the inferior vena cava (black arrow) and the hypodense thrombus (black arrow) around the catheter that prevents hepatic vein drainage. In the sagittal reformat CT image, the distal of the catheter located vena cava inferior is also observed (white arrow, **b**)

He had been diagnosed with familial mediterranean fever 10 years ago and renal biopsy was resulted with AA amyloidosis. After uremic symptoms, hemodialysis was started 3 days a week by transjugular catheter 5 months ago. Because of catheter malfunction, his catheter was replaced in the another medical center. Ten days after catheter malposition, his complaints started. On admission, the patient was good, conscious and orientated and his fever was: 38.5 °C, pulse: 104 beats/minute, respiratory rate: 26/min, blood pressure: 100/50 mmHg. During physical examination, distension, right flank pain and venous collateral development was observed in the patient’s abdomen. Laboratory studies revealed that blood urea nitrogen: 59 (6–20) mg/dl, creatinine: 4.4 (0.5–1.2) mg /dl, sodium: 137 (136–145) mmol /l, potassium: 3,9 (3.5–5.1) mmol/l, Total bilirubin: 1.1 (0–1.4) mg/dl, direct bilirubin: 1.0 (0–0.3) mg/dl, Total protein: 5.1 (6.4–8.3) gr/dL, albumin: 2.4 (3.5–5.2) gr/dl, alkaline phosphatase: 99 (44–130) u / L, − lactate dehydrogenase: 327 (135–250) u/L, aspartate aminotransferase: 142 (0–40) u/L, alanine aminotransferase: 453 (0–41) u /L, gamma glutamyl transferase:: 256, (10–71) u /L, C reactive protein: 174 (0–5) mg /L, procalcitonin: 100 (0–0.5) ng /dl, Hemoglobin: 9.5 (12–16) g /dl, platelet: 65000 (130–400) μ /L, white blood cell: 11 × 10^9^ (4.8–10.7)(10^9^)**/**liter, d dimer: 4220(0–500) ug/L. In the axial contrast enhanced venous phase CT image, a hypodense thrombus was observed in which the distal end of the catheter is at the level of drainage of the hepatic veins into the vena cava inferior and that blocked hepatic vein drainage around the catheter. Additionally, mosaic contrast pattern and perihepatic fluid is seen on computed tomography (CT) (optima CT 540, GE, USA) image (Fig. 1). Thrombus around the catheter was also confirmed by Doppler US (GE Logiq Pg medical System (GE, Healthcare,Chicago,IL,USA))examination.

**Fig. 2:**
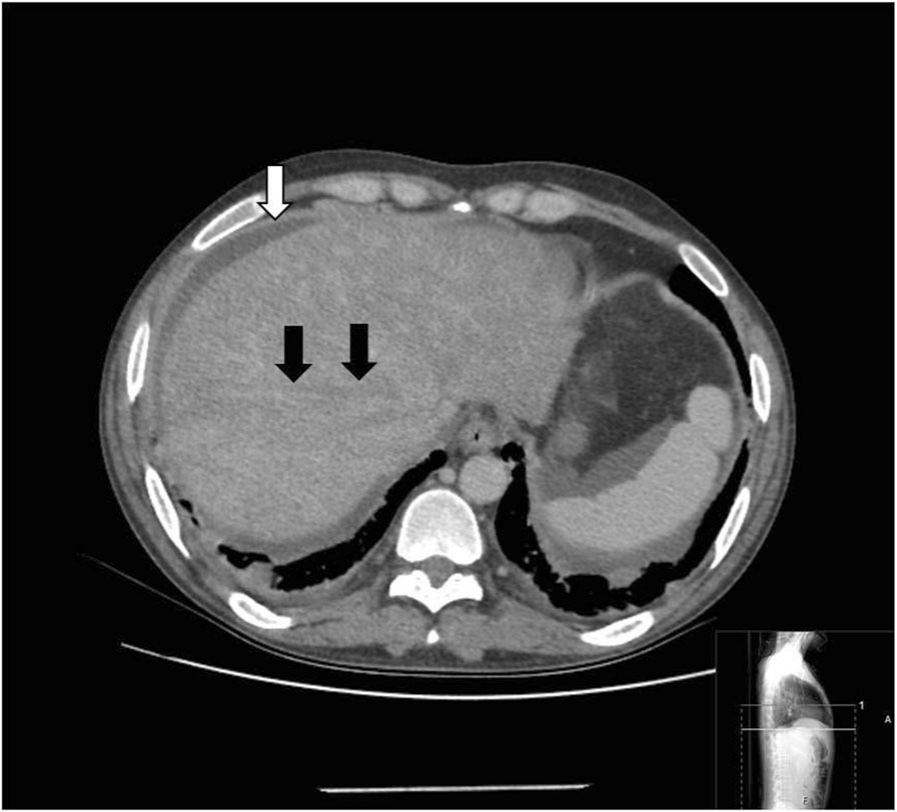
In axial venous phase contrast CT section; Mosaic pattern compatible with Budd-Chiari Syndrome is observed in the liver parenchyma. No contrast agent filling is observed in the hepatic veins (black arrows). In addition, acid is observed in the perihepatic area (white arrow)

The sagittal reformat, in which the distal tip of the catheter was inferior to the vena cava, was also observed in the CT image (Fig. 2) The patient’s diagnosis was accepted Acute Budd Chiari syndrome developed as a result of catheter malposition. Heparin and broad-spectrum antibiotic therapy (Vankomycin/meropenem) were administered for the patient immediately. The catheter was removed and a new catheter was inserted in the same session. A few days after this procedure, symptoms of liver failure regressed (Table 1). Because patient’s general condition was good and without fever, he was discharged with advices on the 9th day of hospitalization.

**Table 1:** Biochemical parameters of patient before and after catheter revision

Laboratory markers	Values		Referance values range	Units (SI)
	Before	After		
Blood urea nitrogen	80	30	6–20	mg/dl
Creatinine	4.4	2.2	0.5–1.2	mg /dl
Sodium	137	135	136–145	mmol /l
Potassium	3,9	3.5	3.5–5.1	mmol/l
Total bilurubin	1.1	0.9	0–1.4	mg/dl
Direct bilurubin	1.0	0.3	0–0.3	mg/dl
Total protein	51	50	64–83	g/dL
Albumin	24	28	35–52	g/L
Alkaline phosphatase	99	95	44–130	u / L
Laktat dehidrogenaz	327	240	135–250	u / L
Aspartate aminotransferase	142	50	0–40	u / L
Alanine aminotransferase	453	40	0–41	u / L
Gamma glutamyl transferase	256	85	10–71	u / L
C reactive protein	174	20	0–5	mg /L
Procalcitonin	100	8	0–0.5	ng /dl,
Hemoglobin	9.5	10	12–16	g /dl
Platelet	65,000	150,000	130–400	μ /L
White blood cell	11 × 10^9^	8 × 10^9^	4.8–10.7 × 10^9^	**/**liter
d dimer	4220	570	0–500	ug/L

## Discussion and conclusion

Tunneled dialysis catheter is still very common in patients with ESRD according to the annual report of the USRDS, it has been reported that it is still most widely used vascular access for the dialysis treatment in the USA [6].

Catheter malposition generally may be related to methodological insufficiency, anatomical variation and operator experience. As a result of these reasons, catheter thrombosis, vascular injury and vascular perforation may ocur in patients [14–19].

Compared to other catheter localizations, all complications associated with the IJV (infection, hematoma, malposition) have 2–5 times less complication rate [10, 11]. Traumatic complications associated with IJV catheter insertion have been well described in the literature, such as Horner’s syndrome (a decreased pupil size, a drooping eyelid and decreased sweating on the affected side of your face with miosis, ptosis and enophthalmos), venous perforation, pneumothorax, and cardiac tamponade [20–23].

Generally, catheter related complications are divided into two as acute and chronic. In one study, hematoma, hemothorax and the lowest rate of catheter malposition were observed among acute complications [10].

After catheter malposition, development of stenosis may occur and the central veins and subsequent obstructive symptoms begin. Diagnosis is usually revealed by imaging such as X-ray, Doppler ultrasonography, CT and angiography. In a 1613 series study by Pickver et al., the malposition rate was found to be 3.3%. In another study, catheter malposition rate was found to be 8.79% of all catheterizations. In a study in which 3213 patients were studied by Napalkov et al., the rate of catheter thrombosis was found to be 0.80% [9, 24].

Budd-Chiari syndrome refers to hepatomegaly that develops as a result of venous thrombotic events, ascites, abdominal swelling and pain. This syndrome is also caused by partial or complete hepatic venous outlet obstruction at either hepatic vein, inferior vena cava (IVC) or right atrium level. In any imaging, enlargement of the caudate lobe, irregularities of the liver contour, intrahepatic collaterals and hypervascular nodules are observed in this syndrome. Etiopathological factors for Budd-Chiari syndrome include various systemic thrombotic and non-thrombotic conditions that can cause venous outflow obstruction in hepatic veins and/or IVC Typically, hypoattenuation echogenicity increases in the central region of the liver due to vascular thrombosis, decrease in periphery [13]. Its main treatment is removal of obstruction, anticoagulant therapy, elimination of the underlying condition and liver transplantation may be required in severe cases [25].

FMF is an autoinflammatory disease and may cause vascular endothelial damage. It has been shown that FMF may predispose to thrombosis with different mechanisms in the literature [26, 27]. In our case, FMF may have contributed catheter induced-thrombosis.

In conclusion, although catheter malposition and thrombosis are not a common complication, it is also important to use imaging methods after the catheterization procedure since they cause serious morbidity and mortality. Therefore, imaging methods should be used during the catheterization process and it should be checked whether it is in appropriate localization after the procedure.

## Data Availability

Data sharing is not applicable.

## Abbreviations

AVF: Arteriovenous fistula
ESRD: End stage renal disease
IVC: Inferior vena cava
IJV: Internal jugular vein
USRDS: United States Renal Data System
